# Selection and Characterization of CSFV-Specific Single-Domain Antibodies and Their Application along with Immunomagnetic Nanobeads and Quantum Dots

**DOI:** 10.1155/2020/3201630

**Published:** 2020-01-30

**Authors:** Shunli Yang, Li Yuan, Youjun Shang, Jinyan Wu, Xiangtao Liu, Jie Zhang, Zygmunt Pejsak, Katarzyna Podgórska, Katarzyna Stepniewska, Muhammad Umar Zafar Khan, Jianping Cai, Shuanghui Yin

**Affiliations:** ^1^State Key Laboratory of Veterinary Etiological Biology, National Foot and Mouth Disease Reference Laboratory, Innovative Team for GI Infection and Mucosal Immunity of Swine and Poultry, Lanzhou Veterinary Research Institute, Chinese Academy of Agricultural Sciences, Lanzhou 730046, China; ^2^Jiangsu Co-Innovation Center for Prevention and Control of Important Animal Infectious Diseases and Zoonoses, Yangzhou 225009, Jiangsu, China; ^3^Department of Swine Diseases, National Veterinary Research Institute, 57 Partyzantow, 24-100 Puławy, Poland

## Abstract

Outbreak of classical swine fever (CSF) results in high mortality and thus causes severe economic losses in the swine industry. Single-domain antibody (sdAb) is the smallest antigen-binding molecule derived from camelid heavy-chain antibodies and has the potential to be used as a molecular probe for detection of CSF virus (CSFV). In this study, two sdAb fragments against the E2 antigen of CSFV were obtained, expressed *in vitro*. The functional characteristics analysis indicated that the recombinant sdAbE2-1 and sdAbE2-2 have excellent binding activity, specificity, and high affinity with equilibrium constant value of 3.34 × 10^−7^ and 1.35 × 10^−8^ M to E2 protein. Then, sdAbE2s were conjugated with quantum dots (QD)/AF488 to synthesize two molecular probes for imaging CSFV distribution in cells. The sdAbE2-1 was also labeled with carboxyl-magnetic beads to construct immunomagnetic nanobeads (IMNBs) able to capture CSFV virions and recombinant E2 protein. QD/AF455-sdAbE2s probes colocalised with CSFV virions in swine testis cells, and IMNBs were used as a detection template and proved to bind specifically with CSFV virions and E2 protein. The selected sdAb fragments and sdAb-based molecular probes may be used for the rapid identification of CSFV during field outbreaks and for research on CSFV and host interactions.

## 1. Introduction

Classical swine fever (CSF), or Hog cholera, is caused by the CSF virus (CSFV), which is on the A-list of OIE Notifiable Diseases. Outbreak of CSF among pigs usually results in high mortality rates and thus causes severe economic losses in the global swine industry [1]. The CSFV genome consists of a single, positive-stranded RNA. It contains a single open reading frame that encodes a polyprotein of 3,898 amino acids. Cleavage of the polyprotein by cellular and viral proteases ultimately yields NH2–N^pro^–C-E^rns^–E1–E2–p7–NS2–NS3–NS4A–NS4B–NS5A–NS5B–COOH [2].

The glycoprotein E2 is present on the envelope of CSFV. It has multiple roles in the CSFV life cycle and participates in viral attachment, cell entry, cell tropism, and virulence. Moreover, it is crucial for inducing host immune response during infection. In pigs, it induces the production of neutralizing antibodies effective against lethal challenge [3–6]. E2 contains four relatively antigenic domains: A, B, C, and D [7]. The E2 protein is the preferred candidate antigen for the development of novel vaccine and diagnostic methods for CSF [8, 9].

The variable domain fragments of camelid immunoglobulin heavy-chain antibodies (HCAbs), also called nanobodies (Nbs) or single-domain antibodies (sdAbs), have been identified and characterized over the past few decades. sdAbs fragments are small (∼15 kDa, 4 nm long and 2.5 nm wide) molecules with a robust structure, high level of stability and solubility, and high affinity and specificity; they are also easily cloned and expressed in vitro [10, 11]. These advantageous properties arise from their single-domain nature and from crucial amino acid mutations in the framework 2 (FR2) region; these mutations render the overall structure of sdAbs more hydrophilic than that of conventional antibody fragments [12]. Their convex surface and extended and more flexible CDR3 loop further enable the recognition of cavities or hidden epitopes on the antigen surface. In addition, sdAbs are nonimmunogenic due to their high similarity to human VH sequences. Thus, they have numerous applications in fundamental research, diagnostics, and therapy [13–16].

In this study, we obtained two single-domain antibodies (sdAbE2s) against the E2 antigen. Both sdAbE2s were derived from *Camelus bactrianus* through phage display technique, expressed in *E. coli* and purified through affinity chromatography. sdAbE2s were labeled with quantum dots (QD)/AF488 to construct two ultrasmall nanoprobes for imaging CSFV in swine testis (ST) cells. sdAbE2-1 was also conjugated with magnetic beads to synthesize functionalized magnetic beads that can specifically capture CSFV and E2 antigen *in vitro*.

## 2. Materials

### 2.1. Ethics Statement

All of the animals were handled in strict accordance with the Animal Ethics Procedures and Guidelines of the People's Republic of China, and the current study was approved by the Animal Ethics Committee of Lanzhou Veterinary Research Institute, Chinese Academy of Agricultural Sciences (No. LVRIAEC2012-006).

### 2.2. *C. bactrianus* Immunization and Library Construction

Truncated E2 protein of CSFV C-strain was expressed in *E. coli* with histidine tag (hrE2) and glutathione S-transferase tag (grE2). A 10-month-old male *C. bactrianus* was immunized with 800 *μ*g recombinant hrE2 and emulsified in 206 oil adjuvant (SEPPIC, France). Serum was isolated prior to immunization and stored as a negative control. On days 21 and 35 after the first immunization, the camel was injected with 500 *μ*g emulsified E2 antigen to boost immunity. Serum samples were collected from the jugular vein after each immunization event for the detection of antibodies through blocking ELISA. Five days after the final immunization, 50 ml of peripheral blood was collected in EDTA-coated tubes from the external jugular vein.

Total RNA was extracted from approximately 1 × 10^10^ peripheral blood lymphocyte cells (PBLC) with Lymphocyte Separation Medium (Solarbio®, Beijing, China), and the first-strand cDNA was synthesized using Superscript II reverse transcriptase with OligodT (18) primer (Invitrogen). HCAb-encoding cDNA was specifically amplified with the first pair of PCR primers [17]. These primers anneal to the leader and CH2 sequences. The 600-bp gene encoding VHH fragments was used as the amplification template for reaction with H2s and H3d, the second pair of PCR primers. These primers anneal at the framework 1 (FR1) and 4 (FR4) regions of VHH. In the last step, VH2 and VH3 primers containing *Sfi*I*/Not*I restriction sites were used. Obtained PCR products were ligated into the phages display system plasmid vector pHEN2 [18] with T4 DNA ligase. After transformation into *E. coli* TG1 cells, VHH library size and diversity were estimated by plating on LB-ampicillin (100 *μ*g/mL ampicillin) agar plates followed by colony PCR using a universal primer. Fifteen positive clones that produced a PCR product of approximately 500 bp were sequenced. The colonies were scraped from the plated with 10 mL 2 × YT-ampicillin (100 *μ*g/mL ampicillin, 1% glucose, and 25% glycerol) and stored at −80°C for further use.

### 2.3. Selection of sdAb Fragments with High Specificity for E2

Three consecutive rounds of phage panning were performed to obtain sdAb clones with high affinity for E2 protein. The grE2 antigen was coated as follows: 100, 50, and 15 *μ*g/mL for rounds 1, 2, and 3, respectively. Blank wells were maintained during panning by coating wells with PBS. The antigen and control wells for all panning rounds were coated with 100 *μ*L well^−1^ and incubated overnight at 4°C. Wells were blocked with block buffer (100 *μ*L, 1% w/v casein in PBS). After phages incubation for 2 h at 37°C, the antigen and control wells were washed 10 times with PBST (0.1% Tween-20 in PBS). For the next two rounds, phages were washed with 0.2% and 0.3% PBST. Bound phages of each round were eluted by adding 100 *μ*L of freshly prepared 100 mM HCl-Glycin (pH 2.2). The eluant was neutralized after incubation for 30 min by adding 100 *μ*L of 1.0 M Tris-HCl (pH 9.1). Output phages were subsequently amplified in exponentially growing *E. coli* TG1 cells. Enrichment factors were calculated on the basis of the input and output phages after each panning round. Individual clones were randomly selected from the three rounds of panning and were subjected to monoclonal phage ELISA to obtain clones that specifically bind to the target antigen [19]. Antigen-reactive clones were sequenced and analyzed for the selection of unique clones.

### 2.4. Structural Simulation

Structural simulation of sdAbE2-1 and -2 was conducted by using online software: SWISS-MODEL (https://swissmodel.expasy.org/). A crystal structure of published sdAb (5lmj) was used as the model [20]. The structure was viewed by SPDBV software version 4.0.

### 2.5. Expression of sdAbE2s in *E. coli* and Cleavage of a SUMO Tag from Recombinant sdAbE2s

sdAb fragments were subcloned into the PE-SUMO expression vector (Lifesensors Inc., UK) double-tagged with SUMO and 6 × His (sdAbE2s: sdAbE2-1, sdAbE2-2) [17]. The expression vector was then transformed into the *E. coli BL21-Codon-Plus (DE3)-RIL* strain (Stratagene). An overnight culture of *E. coli* cells containing recombinant sdAb plasmids in Luria–Bertani (LB) medium (34 *μ*g/ml chloramphenicol and 80 *μ*g/ml kanamycin) were transferred into 0.5 L fresh LB medium at a final concentration of 2.0% and incubated at 37°C until cells reached midlog growth. The remainder of the culture was induced by the addition of isopropyl-thiogalactopyranoside (IPTG) at a final concentration of 0.1 mM and incubated for an additional 22 h at 16°C with shaking at 150 rpm/min. Cells were harvested by centrifugation, and the cell pellet was resuspended in 15 ml of 25 mM Tris-HCl buffer (1.0% Triton X-100, 50 mM Tris-HCl, pH 7.2). Cells were disrupted through ultrasonication on ice and centrifuged at 12000 ×g for 30 min at 4°C. The supernatant was collected, filtered using a 0.45 *μ*m filter, transferred to a column for nickel chelate affinity chromatography, and incubated for 30 min at 4°C. Unbound proteins were removed with Tris-HCl buffer (50 mM imidazole, 50 mM Tris-HCl, pH 7.2) with Bradford reagent (v/v: 5% of 95% ethanol, 10% of 88% phosphoric acid, and 0.07 mg/ml Coomassie brilliant blue G-250) until the protein indicator no longer turned blue. Bound protein was eluted with Tris-HCl buffer (300 mM imidazole, 150 mM NaCl, and 50 mM Tris-HCl, pH 7.2) until the protein indicator could no longer turn blue. The amount of purified sdAb-tag protein was quantified by Nanodrop 2000 spectrophotometer (Thermo, USA). Purified proteins (5 *μ*g) were analyzed by 12% SDS-PAGE and evaluated by Western blotting (WB) analysis based on 1 : 1000 diluted HRP-conjugated anti-His mouse monoclonal antibody (Kangwei, China). The ECL substrate (Thermofisher, USA) was used for the chemiluminescence reaction. Purified proteins were stored at 4°C for further analysis.

A cleavage reaction assay was performed as previously described [17]. Briefly, 200 *μ*g of fusion sdAbE2 protein and 50 units of Sumo protease were incubated with protease buffer (50 mM Tris-HCl, 0.2% Igepal, and 1 mM DTT, pH 7.2) were added to the solution at 16°C overnight. The mixture was diluted with binding buffer (50 mM Tris-HCl, 150 mM NaCl and 30 mM imidazole, pH 7.5) and mixed with 500 *μ*l resin for the removal of protease, His-tag, and redundant fusion sdAbE2s. The purified tagless sdAbE2 protein was identified through 12% SDS-PAGE.

### 2.6. Determination of the Binding Affinity and Specificity of Recombinant sdAbE2s

The wells of microplate were coated with grE2 protein and then incubated serial dilutions of sdAbE2s (10, 5, 2.5, 1.25, 0.63, 0.32 0.16, and 0 *μ*g/ml) to further assess the binding capacity of recombinant sdAbE2s with the grE2 protein. The proteins were then subjected to indirect ELISA with HRP-conjugated anti-His-antibody as the secondary antibody. For WB, grE2 protein was transferred to a film and incubated with two sdAbE2s and polyclonal serum against CSFV obtained from the infected pig. Meanwhile, CSFV infected ST cells were lysed. The lysates were separated by SDS-PAGE and transferred to a film. sdAbE2s, porcine polyclonal serum against CSFV, and negative serum were incubated with the film, respectively. The HRP-conjugated anti-His-antibody and antiporcine IgG antibody were used as the secondary antibody.

The binding affinity of sdAbE2s to grE2 protein was evaluated by surface plasmon resonance (SPR) using Biacore 3000 as described previously [19]. Briefly, the carboxymethyl dextran surface of research-grade CM5 (GE healthcare) chips was activated and coated with recombinant grE2 protein to 3000 resonance units (RU). The serially diluted sdAbE2s was injected over the E2 surfaces for 180 s at 10 *μ*L/min, respectively. All SPR assays were under the condition of 25°C. Association and dissociation rate constants (Ka and Kd) were calculated using BIAcore evaluation 4.1 software.

The specificity of recombinant sdAbE2s was examined and evaluated through indirect ELISA using the recombinant CSFV grE2 structural protein as the positive control as well as porcine circovirus type 2 (PCV2) Cap, porcine reproductive and respiratory syndrome virus (PRRSV) GP5 [21], foot-and-mouth disease virus (FMDV) VP1 (own production), and bovine viral diarrhoea virus (BVDV) E2 (own production) proteins. Antigens (10 *μ*g/mL) were coated at 100 *μ*l/well in carbonate-buffered saline (pH 9.6) and stored overnight at 4°C. The wells were washed four times with 200 *μ*l/well 1 × PBST and blocked for 45 min with 2% gelatine in 1 × PBST. Thereafter, 1.53 *μ*g/ml of sdAbE2s was incubated with antigens at 37°C for 1 h and then with HRP-anti-His monoclonal antibody. The TMB reagent was used to develop signals.

The specificity of sdAbE2s was evaluated using triplicate samples of each sdAb and a commercially available blocking ELISA kit (HerdChek, CSFV Ab, IDEXX Laboratories, USA) in accordance with the manufacturer's instructions. The blocking rate was then calculated.

### 2.7. sdAbE2s Bind to Overlapping Epitopes

Binding to an individual or overlapping epitopes was assessed through ELISA to test whether sdAbE2s recognize the same antigenic site [22]. grE2 was coated at a concentration of 2.35 *μ*g/mL (100 *μ*L/well) and incubated overnight at 4°C. After blocking with 1% bull serum albumin (Sigma, USA), 2 *μ*g of sdAbE2s were added individually and in pairs at the concentration sufficient to saturate the coated antigen (1.26 *μ*g). After incubation and washing, the bound antibody was detected through the addition of HRP-anti-His mouse monoclonal antibody. Additivity index (AI) was determined by the formula

(1) $$\begin{array}{c} \text{A}.\text{I}.=\left[\frac{2A_{\left(1+2\right)}}{A_{1}+A_{2}}-1\right]\times 100, \end{array}$$

where *A*_1_, *A*_2_, and *A*_(1+2)_ are OD_490_ nm values obtained in ELISA, with the first sdAb alone, the second sdAb alone, and the two sdAb together. If the value of A.I. more than 40%, suggesting the two antibodies are bind to different epitopes.

### 2.8. Production of Immunomagnetic Nanobeads for Binding with CSFV and grE2 Protein

Immunomagnetic nanobeads (IMNBs) were prepared by incubation of superparamagnetic nanobeads (200 nm, 3 mg/ml, purchased from Ademtech SA, Pessac, France) activated by 1-ethyl-3-[3-dimethyl aminopropyl] carbodiimide hydrochloride and N-hydroxy succinimide with 1 mg/ml of sdAbE2-1 in 1 ml of 0.01 M phosphate-buffered saline (PBS, pH 7.2) for 4 h at 37°C with gentle shaking at 120 rpm. The beads were then blocked with 0.1% (m/v) bovine serum albumin (BSA) for 30 min at 37°C. IMNBs were separated with a magnetic scaffold and resuspended in 1 ml of PBS and stored at 4°C. Then, IMNBs (20 *μ*l, 3 mg/ml) were incubated with 1000, 500, 400, 300, 200, and 100 *μ*l of CSFV (5,000RID, C-strain) propagated in ST cell culture and grE2 protein (0.5 mg/ml), respectively, at 37°C for 1 h. IMNB-CSFV and IMNB-grE2 composites were then separated from the suspension with a magnetic scaffold and washed five times with PBST (0.5% Tween-20 in PBS, pH 7.2). IMNB-CSFV complexes were used as a template for total RNA extraction using RNA Extraction Kit (QIAGEN, Hilden, Germany) for the amplification of the E2 gene by RT-PCR. IMNB-grE2 was subjected to SDS-PAGE and WB.

### 2.9. QD605-Labeled sdAbE2-1 and AF488-Labeled sdAbE2-2 as Imaging Probes for CSFV in ST Cells

Activated QD_605nm_ (ZnS-capped CdSe) was modified with thioglycolic acid and then subjected to reaction with 1 mg of sdAbE2-1 to obtain the QD-sdAbE2-1 probe following a previously described protocol [23, 24]. AF488 (Alexa Fluor 488, Wuhan Jiayuan QuantumDots Co. Wuhan, China) was conjugated with sdAb-E2-2 forming AF488-sdAbE2-2. The probes were stored at 4°C for the determination of specificity against CSFV in ST cell culture. Monolayers of ST cells were cultured on coverslips in six-well plates and infected with the C-strain of 5,000RID CSFV. After 2 h of adsorption, the supernatant was removed, and the monolayer was washed twice with fresh medium. The cells were incubated in medium containing 0.5% fetal bovine serum at 37°C with 5% CO_2_. At 24 h postinfection (h.p.i.), cells on coverslips were fixed with 4% paraformaldehyde for 10 min and then permeabilized with 0.5% Triton X-100 (Sigma, USA) for 20 min at 37°C. The cells were incubated with the QD/AF488–sdAbE2s probes (diluted in PBS) overnight at 4°C with gentle shaking and then washed five times with PBST buffer. Cell nuclei were stained with 4,6-diamidino-2-phenylindole (DAPI) solution for 8 minutes at room temperature. Images were visualized using a laser scanning confocal microscope.

## 3. Results

### 3.1. sdAbE2s Library Construction, Screening, and VHH Fragment Analysis

E2 protein of CSFV was recombinantly expressed in E. coli with His-tag (hrE2) and GST-tag (grE2) ([Supplementary-material supplementary-material-1]). The immune reaction against the E2 antigen induced in *C. bactrianus* was monitored and evaluated through ELISA. The results showed that seroconversion occurred after the first immunization event. The PBLC fraction was collected five days after the last immunization, and mononuclear cells were isolated, harvested, and stored in liquid nitrogen.

Three rounds of PCR were performed using different primer pairs to amplify the coding fragments of VH and VHH from the cDNA template. The PCR product obtained based on the third pair of PCR primers, which contained *Sfi*I/*Not*I restriction sites and was 450 bp in size, was ligated with the phagemid vector pHEN2 and transformed into *E. coli* TG. The antibody library of sdAb was generated by scraping the clones from the plated cells with 2 × YT-ampicillin. Colony screening by PCR showed that 26 out of 30 of the clones (86.67%) contained a plasmid with an insertion of the expected size for camel sdAb gene. The capacity of the library was 10^6^ of that calculated by the positive clone numbers. A total of 2.02 × 10^4^ colonies were harvested from the dilution plating of the cultured library by polyclonal phage ELISA using the coated recombinant E2 antigen (Table 1). Forty clones from the third elution were subjected to single-phage ELISA to bind the target E2 antigen. Two unique sdAb genes fragments were observed through sequence alignment and were designated as sdAbE2-1 and sdAbE2-2.

### 3.2. Analysis of sdAb Sequences

**Figure 1:**
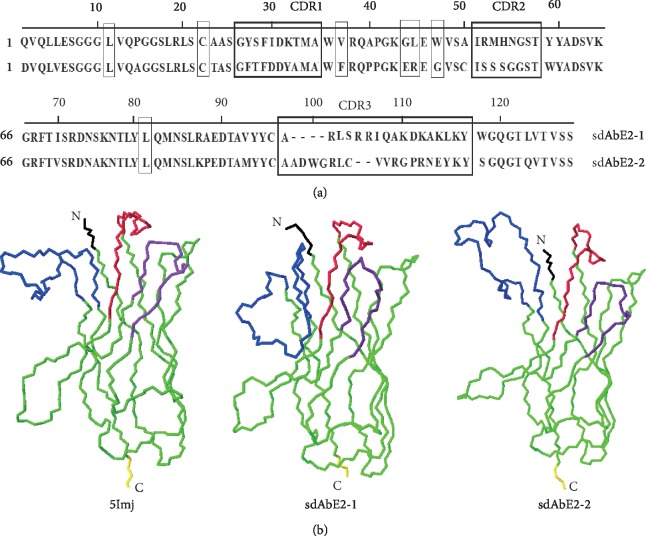
Alignment and homology modeling of specific sdAbE2s. (a) The amino acid sequences were aligned according to the Kabat numbering. The hallmark amino acids of sdAbs were dotted boxed and CDRs were grey-boxed, and the Cysteines were underlined. The CDR3 has lengths of 17 and 19 amino acids. (b) Homology modeling of sdAbE2-1 and sdAbE2-2 by SWISS-MODEL workspace. A published sdAb (5lmj) which show high identity with sdAbE2s was used as the model. CDR1, CDR2, and CDR3 of sdAbE2s and 5lmj are represented in red, purple, and, blue, respectively. The N- and C-terminal extremities are labeled N and (c), respectively. This figure was generated by SPDBV software.

Phage display was used to select two specific sdAb antibodies against CSFV E2 protein from the immunized camel. Analysis of amino acid fragments showed that the hallmark residues Phe37, Glu44, Arg45, Gly47, and Leu81 in sdAbE2-2 were consistent with those in previous reports [25, 26] but sdAbE2-1 had some differences. CDR3 in sdAdE2-1 had a length of 17 and sdAdE2-2 of 19 amino acids. Cysteine-based disulfide bond plays an important role in the stabilities of sdAb [27]. One pair of cysteines Cys22 and Cys96 were present in sdAdE2-1, and two pairs of cysteines Cys22 and Cys96, Cys50 and Cys104 were present in sdAdE2-2 (Figure 1(a)).

Proposed structures for sdAbE2-1 and sdAbE2-2 were obtained by modeling them on the known structure of 5mlj, a nanobody with a high degree of sequence identity 72.5% and 80.3% to these nanobodies, respectively (Figure 1(b)).

### 3.3. Expression, Purification, and WB Analysis of Recombinant sdAbE2s

**Figure 2:**
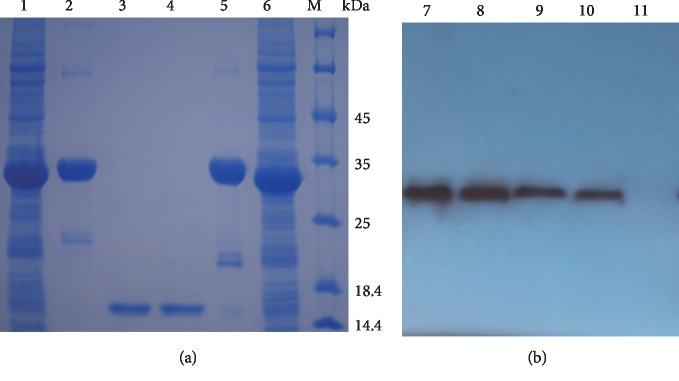
Identification of recombinant sdAbE2s by SDS-PAGE and Western blot. (a) Analysis of expression sdAbE2 with SDS-PAGE. Lane M protein MW marker; lanes 1 and 6 were the expression groups of sdAbE2-1 and sdAbE2-2 induced with IPTG. Lanes 2 and 5 were the Ni-NTA-affinity purified sdAbE2-1 and sdAbE2-2. Lanes 3 and 4 were purified products of sdAbE2-1 and sdAbE2-2 by the cleavage of fusion tag of SUMO and 6 × His tag. (b) Verification by Western blotting. Western blotting was performed using HRP-anti-His mouse monoclonal antibody and ECL substrate. Lanes 7 and 8 were the lysates of expression group and purification group of sdAbE2-1. Lanes 9 and 10 were the lysates of expression group and purification group of sdAbE2-2. Lane 11 uninduced control group.

The sdAbE2s were expressed in *E. coli* and purified through nickel affinity chromatography. 12% SDS-PAGE analysis revealed that the purified sdAbE2s and tagless sdAbE2 had the expected molecular weights of 35 and 14 kDa, respectively (Figure 2(a)). Moreover, the expression and purification of sdAbE2s were evaluated via WB analysis, and specific bands, shown the same molecular weight with SDS-PAGE, were observed (Figure 2(b)).

### 3.4. Characterization of Recombinant sdAbE2s

**Figure 3:**
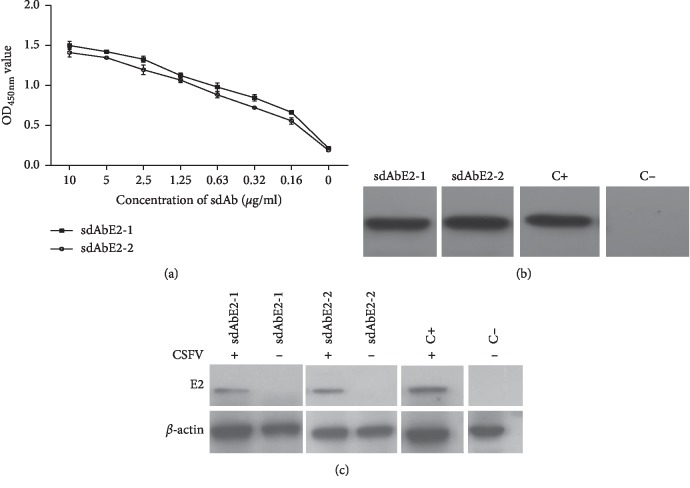
Characterization of recombinant sdAbE2s by indirect ELISA and Western blot. Binding activity of sdAbE2 with grE2 protein was analyzed by indirect ELISA and western blot. (a) A dilutions series of each sdAbE2s were used for detection grE2 protein by indirect ELISA with HRP conjugated anti-HIS-antibody as the secondary antibody. (b) The analysis of sdAbE2s against grE2 binding capacity by western blot. CSFV immune serum and preimmune serum were used as positive (lane C+) and negative control (lane C-). (c) The analysis of sdAbE2s against E2 of CSFV in ST cells by western blot. *β*-actin was used as an internal reference protein. Error bars represent ±SD of three independent measurements.

Indirect ELISA showed that OD_450_ values decreased with decreasing sdAbE2s concentration (Figure 3(a)) and that sdAbE2s can recognize the grE2 antigen. WB revealed that sdAbE2s can bind to E2 similarly as the polyclonal serum against CSFV (Figures 3(b) and 3(c)). To further assess the ability of selected sdAbE2s to bind to the E2 protein, binding kinetics were tested using SPR. sdAbE2-1 and sdAbE2-2 showed the KD value of 3.34 × 10^−7^ M and 1.35 × 10^−8^ M, respectively (Table 2).

**Figure 4:**
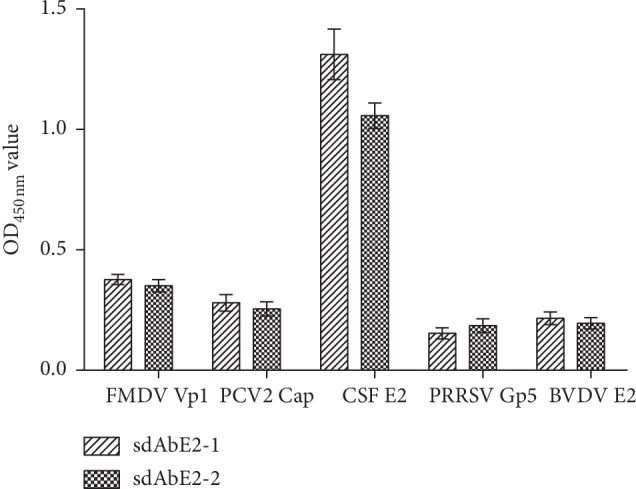
The cross-reaction assay of recombinant sdAbE2s by indirect ELISA. The wells coated with recombinant PCV2 Cap, PRRSV GP5, FMDV VP1, and BVDV E2 antigens served as test samples, the grE2 was used as a positive control. Error bars represent the ±SD of three independent measurements.

The specificity of sdAbE2s was validated through indirect ELISA. The cross-reaction assay showed that both sdAbE2s can specifically bind with the grE2 protein of CSFV and do not cross-react with PCV2 Cap, PRRSV GP5, FMDV VP1, or BVDV E2 proteins (Figure 4). Two sdAbE2s targeting E2 protein were tested through the ELISA additivity experiment. The measured additivity index (AI%) of sdAbE2-1 with sdAbE2-2 was 65.25%, indicating that the two sdAbE2s bind to different epitopes on the E2 protein (Table 3). Blocking ELISA showed that sdAbE2-1 and sdAbE2-2 had blocking rates of 77% and 59%, respectively. These data suggested that the two sdAbE2s are highly specific for the E2 antigen.

### 3.5. Capture Activity of sdAbE2 Based Immunomagnetic Beads

**Figure 5:**
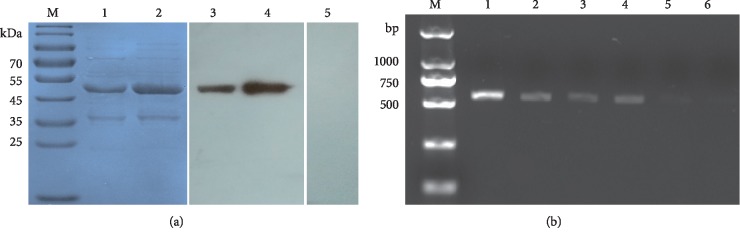
Capture of IMNBs to CSF virus and grE2 protein. Lysates of CSFV (C-strain) infected ST cells and grE2 protein were incubated with IMNBs at 37°C for 1 h. (a) The captured grE2 protein by IMbeads (Lane 1) and total used grE2 protein (Lane 2) were visualized by SDSP-PAGE and confirmed by western blot (Lane 3 and 4). (b) Detection of the binding to CSFV in IMNBs. 1000, 500, 400, 300, 200, and 100 *μ*l of CSFV (Lanes 1 to 6) propagated in ST cell culture were incubated with IMNBs and the size of 560 bp E2 gene was amplified by RT-PCR.

sdAbE2-1 was coated onto magnetic beads in order to specifically bind and enrich CSFV and grE2 from cell culture supernatants of CFSV-infected cells and recombinant grE2, respectively. Total viral RNA was extracted from IMNB–CSFV and amplified through RT-PCR. The 560-bp E2 fragment of CSFV was observed, and results indicated a linear relationship between viral titers and the brightness of target bands (Figure 5(b)). The results of SDS-PAGE and WB assay indicated that magnetic beads coated with sdAbE2-1 were able to bind recombinant grE2 protein in solution (Figure 5(a)).

### 3.6. CSFV Imaging in ST Cells with sdAbE2s Probe

**Figure 6:**
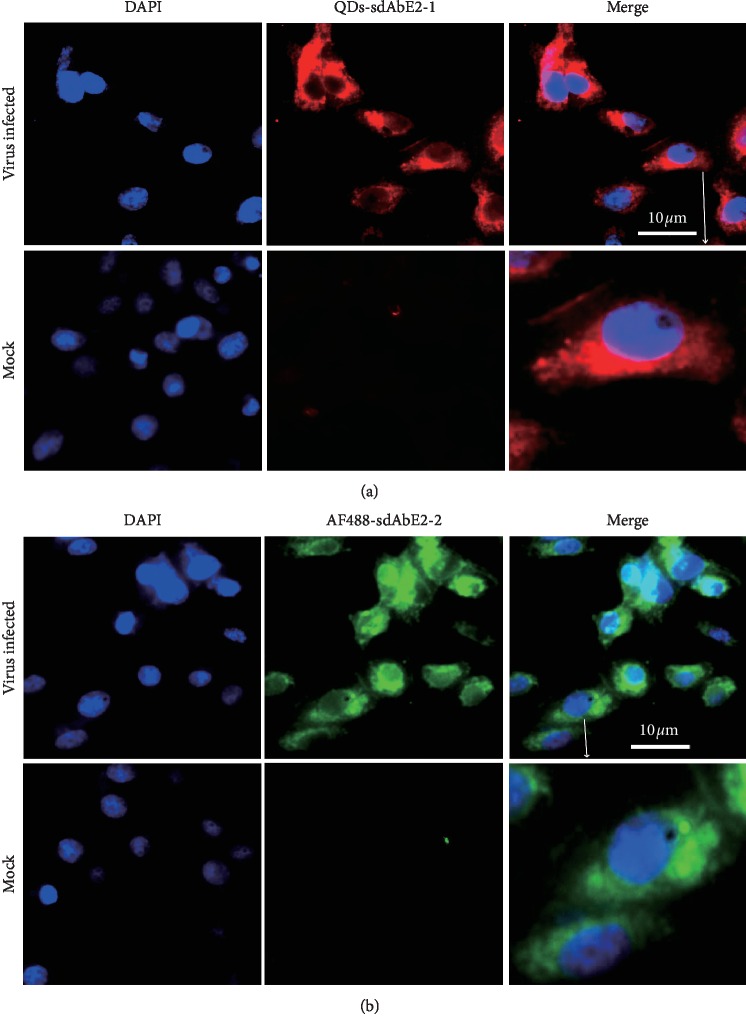
QDs605/AF488 labeled sdAbE2s as probes imaging CSFV in ST cells. The ST cells were harvested at 24 h.p.i and processed for immunofluorescence with AF488-sdAbE2-2 and QDs-sdAbE2-1. All nuclei were stained with DAPI. The merged images show the colocalization of CSFV with different probes.

ST cell culture infected with CFSV was fixed at 24 h.p.i and the presence of CSFV virions was detected through direct immunofluorescence assay (DIFA). Reaction with QD-sdAbE2-1 and AF488-sdAbE2-2 probes revealed that the majority of CSFV concentrated around the nuclei. In particular, the level of fluorescence proportional to the number of viral particles was higher in areas surrounding condensed nuclei compared to normal nuclei (Figure 6). This result indicated that the QD-sdAbE2-1 and AF488sdAbE2-2 probes have potential applications in research on CSFV-host interactions.

## 4. Discussion

Specific antibodies are essential tools in many diagnostic techniques and tests, including ELISA, flow cytometry, western blotting, and immunofluorescence, for detection of molecular targets. However, conventional antibodies are frequently difficult to produce because of their complex structure. Small molecular antibodies like single-chain Fv (scFv), Fab, and sdAbs show an attraction towards natural antibodies. In particular, an advantage of small antibody fragments is their ease of genetic manipulation due to their smaller size, ease of expression in bacterial systems, easy adaptation to high-throughput screening *in vitro*, and in scaled-up production, and easy to be purified and labeled [28, 29]. These properties and highlight allow them to be used widely in biomedicine and biotechnology. Over the decades, sdAbs, more frequently referred to be as nanobodies, has been described as a credible next-generation antibody-derived biologics with significant as advances over conventional antibodies [30]. The sdAbs produced by *Camelidae* that has only one variable region (heavy chain), with a reduced size, and show greater stability under a range of conditions for medical and technological applications [12, 31]. In contrast to McAb, lymphocytes from the PBMC pool of immunized camels should be a good source of Ag-binding sdAb fragments for the construction of a large immune library. Immune library construction does not harm the experimental animal given that only a small amount of blood is collected throughout the procedure. Recombinant sdAbs with high affinity, thermal stability, solubility, and specificity can be produced *in vitro* with different libraries [32, 33]. This production method can be easily scaled up for the cost-effective production of monoclonal antibodies.

E2 is the most immunogenic protein of CSFV and induces the production of neutralizing antibodies against lethal challenges. Although CSFV forms a single serotype [7, 34], molecular epidemiology studies have classified CSFV strains into three groups on the basis of the E2 gene sequence [35, 36]. The antigenic domains A, B, C, and D in the N-terminal half of E2 are highly stable and conserved. These domains induce the expression of protective neutralizing antibodies. Thus, the E2 gene of CSFV is the best candidate target for recombinant vaccine development and specific antibody production. In this study, recombinant E2 of CSFV was used as an antigen for immunization of camel and screening of sdAbs from the immune library.

After three rounds of panning, two clones showing different sequences were selected. The amino acid sequences of sdAbE2-2 showed characteristic substitutions at four positions in FR2, but sdAbE2-1 did not show that [25]. However, sdAbE2-1 showed similar length in CDRs which contribute to the character of the high affinity of sdAbs [37]. In addition, both sdAbE2-1 and sdAbE2-2 were able to soluble expressed in *E. coli*. Thus, sdAbE2-1 was also a usable binder.

To evaluate the features of sdAbE2s, the binding activity, affinity, and specificity to E2 protein were analyzed. ELISA and Western blot assay indicated that sdAbE2-1 and sdAbE2-2 display excellent binding activity to E2 protein. Further assessment for the affinity by SPR technique showed that sdAbE2-1 and sdAbE2-2 unfold equilibrium dissociation constant value of 3.34 × 10^−7^ and 1.35 × 10^−8^ M to E2 protein that was similar with the published sdAbs [38]. However, both sdAbE2-1 and sdAbE2-2 do not show obvious neutralizing activity to CSFV C-strain ([Supplementary-material supplementary-material-1]), and only has the ability of specific binding the E2 protein and CSFV. Hence, they will be used to develop diagnostic products on CSFV.

Functionalized magnetic nanoparticles are successfully applied in bioseparation and detection for viruses [24, 39, 40]. Initial results obtained for sdAbsE2s conjugated with immunomagnetic nanobeads (IMNBs) presented in this study confirmed their usefulness in antigen detection and enrichment methods. QDs-antibody conjugates are widely used for in vitro diagnostics and labeling cell-surface antigens. Alternatively, primary antibodies can be used with secondary antibodies conjugated to a QD. A wide range of conjugation methods for binding QDs to biological molecules has been reported [41]. There are many well-established protocols for the conjugation of high-specificity and high-affinity mAbs or targets to the surface of water-soluble QDs in order to obtain bright labels [24, 42, 43]. In the current study, we conjugated sdAb fragments with QDs and AF488 to synthesize the nanoprobes, which were used for imaging CSFV in ST cells in a direct immunofluorescence assay. The observed fluorescence in ST cells nicely reflected that CSFV can be recognized by the QDs/AF488-sdAb probe. As a diagnostic reagent, the QDs/AF488-sdAb probe used in a direct immunofluorescence assay showed high efficiency with lesser steps and shorter times compared with indirect immunofluorescence assay.

The range of possible applications of sdAb has extensively expanded. Custom affinity chromatography for the purification of bioactive therapeutics, prothrombin, and a recombinant immunotoxin has been designed and developed using camelid VHH antibody fragments as “tunable” immunoaffinity ligands [44]. The small size of nanobodies can facilitate the early diagnosis and prevention of cancer by detecting or defining cancer biomarkers, such as AFP, CAIX, PMSA, TAG-72, or HER2 [13]. The selected sdAbE2s have the potential application as a diagnostic reagent for effective detection of CSFV and as a research tool in CSFV-host interactions. Additionally, the selected sdAbE2s are a useful resource for the further discovery of more sdAb properties.

## 5. Conclusion

In conclusion, we have successfully obtained two sdAbE2s against E2 protein from the immunized *C. bactrianus* sdAb library by phage display technique. The functional features analysis revealed that the recombinant sdAbE2-1 and sdAbE2-2 have excellent binding activity, specificity, and high affinity with equilibrium constant value of 3.34 × 10^−7^ and 1.35 × 10^−8^ M to E2 protein, but have no neutralizing activity to CSFV. The successful detection of CSFV based on IMNB, QDs-sdAbE2-1, and AF488-sdAbE2-2 clearly reflect that CSFV virions can be reliably recognized by sdAbE2s. These results suggested that the two selected recombinant sdAbE2s have potential applications for the rapid identification of CSFV and in basic research on CSFV with host interactions.

The sdAb library was subjected to three rounds of panning. Input and output phages were quantified by serial dilution with PBS and plating on LB-ampicillin agar plate. a: enriching factor was calculated by the computational formula (output/input). b: the control well with no E2 antigen coated.

## Figures and Tables

**Table 1:** Process monitoring of the panning.

Round	Input	Output	Enriching factor^a^
1	1.60 × 10^11^	4.88 × 10^3^	3.28 × 10^7^
2	6.0 × 10^10^	8.52 × 10^5^	7.04 × 10^4^
2-N^b^	2.0 × 10^10^	7.7 × 10^4^	2.60 × 10^5^
3	1.59 × 10^10^	1.31 × 10^7^	1.21 × 10^4^
3-N	5.3 × 10^10^	2.62 × 10^4^	2.02 × 10^4^

**Table 2:** Kinetics sdAbE2s interaction with E2 protein.

Clones	Ka (M^−1^·s^−1^)	Kd (s^−1^)	KD (M)
SdAbE2-1	2.37 × 10^4^	7.91 × 10 ^−3^	3.34 × 10 ^−7^
SdAbE2-2	6.35 × 10^5^	8.59 × 10 ^−3^	1.35 × 10 ^−8^

ka, association constant; kd, dissociation constant; KD, equilibrium dissociation constant (KD = kd/ka).

**Table 3:** Measured values of the ELISA additivity experiment.

VHHs	sdAbE2-1		sdAbE2-2	
	OD_450nm_ value	AI (%)	OD_450nm_ value	AI (%)
sdAbE2-1	0.765 ± 0.087	—	1.246 ± 0.064	65.25
sdAbE2-2	1.246 ± 0.064	65.25	0.743 ± 0.086	—

Additivity index AI (%) = [(2*A*_(1+2)_/*A*_1_ + *A*_2_) − 1] × 100. The value was the average of three wells ±SD.

## Data Availability

The data used to support the findings of this study are available from the corresponding author upon request.

## Supplementary Materials

Supplementary Materials Supplementary Figure 1: expression of E2 protein with different tag proteins that were used for the selection and evaluation of single-domain antibody. Supplementary Figure 2: analysis of neutralizing activity of sdAbE2s.

## References

[1] Moennig V. (2000). Introduction to classical swine fever: virus, disease and control policy. *Veterinary Microbiology*.

[2] Meyers G., Thiel H. J., Rümenapf T. (1996). Classical swine fever virus: recovery of infectious viruses from cDNA constructs and generation of recombinant cytopathogenic defective interfering particles. *Journal of Virology*.

[3] Wang Z., Nie Y., Wang P., Ding M., Deng H. (2004). Characterization of classical swine fever virus entry by using pseudotyped viruses: E1 and E2 are sufficient to mediate viral entry. *Virology*.

[4] Reimann I., Depner K., Trapp S., Beer M. (2004). An avirulent chimeric pestivirus with altered cell tropism protects pigs against lethal infection with classical swine fever virus. *Virology*.

[5] Risatti G. R., Borca M. V., Kutish G. F. (2005). The E2 glycoprotein of classical swine fever virus is a virulence determinant in swine. *Journal of Virology*.

[6] König M., Lengsfeld T., Pauly T., Stark R., Thiel H. J. (1995). Classical swine fever virus: independent induction of protective immunity by two structural glycoproteins. *Journal of Virology*.

[7] Wensvoort G., Terpstra C., de Kluijver E. P., Kragten C., Warnaar J. C. (1989). Antigenic differentiation of pestivirus strains with monoclonal antibodies against hog cholera virus. *Veterinary Microbiology*.

[8] Bouma A., de Smit A. J., de Kluijver E. P., Terpstra C., Moormann R. J. M. (1999). Efficacy and stability of a subunit vaccine based on glycoprotein E2 of classical swine fever virus. *Veterinary Microbiology*.

[9] Qi Y., Zhang B.-Q., Shen Z., Chen Y.-H. (2009). Candidate vaccine focused on a classical swine fever virus epitope induced antibodies with neutralizing activity. *Viral Immunology*.

[10] Hamers-Casterman C., Atarhouch T., Muyldermans S. (1993). Naturally occurring antibodies devoid of light chains. *Nature*.

[11] Muyldermans S. (2001). Single domain camel antibodies: current status. *Reviews in Molecular Biotechnology*.

[12] Harmsen M. M., De Haard H. J. (2007). Properties, production, and applications of camelid single-domain antibody fragments. *Applied Microbiology and Biotechnology*.

[13] Kijanka M., Dorresteijn B., Oliveira S., van Bergen en Henegouwen P. M., Henegouwen P. M. (2015). Nanobody-based cancer therapy of solid tumors. *Nanomedicine*.

[14] Siontorou C. G. (2013). Nanobodies as novel agents for disease diagnosis and therapy. *International Journal of Nanomedicine*.

[15] Chakravarty R., Goel S., Cai W. (2014). Nanobody: the “magic bullet” for molecular imaging?. *Theranostics*.

[16] De Meyer T., Muyldermans S., Depicker A. (2014). Nanobody-based products as research and diagnostic tools. *Trends in Biotechnology*.

[17] Yin S., Sun S., Yang S., Shang Y., Cai X., Liu X. (2010). Self-assembly of virus-like particles of porcine circovirus type 2 capsid protein expressed from *Escherichia coli*. *Virology Journal*.

[18] Moutel S., Bery N., Bernard V. (2016). NaLi-H1: a universal synthetic library of humanized nanobodies providing highly functional antibodies and intrabodies. *eLife*.

[19] Yang S., Shang Y., Yin S. (2014). Selection and identification of single-domain antibody fragment against capsid protein of porcine circovirus type 2 (PCV2) from *C. bactrianus*. *Veterinary Immunology and Immunopathology*.

[20] Duhoo Y., Roche J., Trinh T. T. N. (2017). Camelid nanobodies used as crystallization chaperones for different constructs of PorM, a component of the type IX secretion system from Porphyromonas gingivalis. *Acta Crystallographica Section F, Structural Biology Communications*.

[21] Yang S., Shang Y., Wang D., Yin S., Cai J., Liu X. (2015). Diagnosis of porcine circovirus type 2 infection with a combination of immunomagnetic beads, single-domain antibody, and fluorescent quantum dot probes. *Archives of Virology*.

[22] Friguet B., Djavadi-Ohaniance L., Pages J., Bussard A., Goldberg M. (1983). A convenient enzyme-linked immunosorbent assay for testing whether monoclonal antibodies recognize the same antigenic site. Application to hybridomas specific for the β2-subunit of Escherichia coli tryptophan synthase. *Journal of Immunological Methods*.

[23] Wang D., Yang S., Yin S. (2015). Characterization of single-domain antibodies against foot and mouth disease virus (FMDV) serotype O from a camelid and imaging of FMDV in baby hamster kidney-21 cells with single-domain antibody-quantum dots probes. *BMC Veterinary Research*.

[24] Yang S., Li L., Yin S. (2018). Single-domain antibodies as promising experimental tools in imaging and isolation of porcine epidemic diarrhea virus. *Applied Microbiology and Biotechnology*.

[25] Muyldermans S., Atarhouch T., Saldanha J., Barbosa J. A. R. G., Hamers R. (1994). Sequence and structure of VH domain from naturally occurring camel heavy chain immunoglobulins lacking light chains. *Protein Engineering, Design and Selection*.

[26] Vu K. B., Ghahroudi M. A., Wyns L., Muyldermans S. (1997). Comparison of llama VH sequences from conventional and heavy chain antibodies. *Molecular Immunology*.

[27] Zhang B., Yu J., Liu C. (2016). Improving detection sensitivity by oriented bioconjugation of antibodies to quantum dots with a flexible spacer arm for immunoassay. *RSC Advances*.

[28] Hoogenboom H. R. (2005). Selecting and screening recombinant antibody libraries. *Nature Biotechnology*.

[29] Ahmad Z. A., Yeap S. K., Ali A. M., Ho W. Y., Alitheen N. B., Hamid M. (2012). scFv antibody: principles and clinical application. *Clinical & Developmental Immunology*.

[30] Wang Y., Fan Z., Shao L. (2016). Nanobody-derived nanobiotechnology tool kits for diverse biomedical and biotechnology applications. *International Journal of Nanomedicine*.

[31] Gonzalez-Sapienza G., Rossotti M. A., Tabares-da Rosa S. (2017). Single-domain antibodies as versatile affinity reagents for analytical and diagnostic applications. *Frontiers in Immunology*.

[32] Leow C. H., Fischer K., Leow C. Y., Cheng Q., Chuah C., McCarthy J. (2017). Single domain antibodies as new biomarker detectors. *Diagnostics*.

[33] Anderson G. P., Liu J. H., Zabetakis D., Liu J. L., Goldman E. R. (2017). Thermal stabilization of anti-*α*-cobratoxin single domain antibodies. *Toxicon*.

[34] Liao X., Wang Z., Cao T. (2016). Hypervariable antigenic region 1 of classical swine fever virus E2 protein impacts antibody neutralization. *Vaccine*.

[35] Paton D. J., McGoldrick A., Greiser-Wilke I. (2000). Genetic typing of classical swine fever virus. *Veterinary Microbiology*.

[36] Sun S.-Q., Yin S.-H., Guo H.-C., Jin Y., Shang Y.-J., Liu X.-T. (2013). Genetic typing of classical swine fever virus isolates from China. *Transboundary and Emerging Diseases*.

[37] De Genst E., Silence K., Decanniere K. (2006). Molecular basis for the preferential cleft recognition by dromedary heavy-chain antibodies. *Proceedings of the National Academy of Sciences*.

[38] Li T., Bourgeois J.-P., Celli S. (2012). Cell-penetrating anti-GFAP VHH and corresponding fluorescent fusion protein VHH-GFP spontaneously cross the blood-brain barrier and specifically recognize astrocytes: application to brain imaging. *The FASEB Journal*.

[39] Lafaye W., Zhang W.-P., Zhang Z.-L. (2012). Robust and highly sensitive fluorescence approach for point-of-care virus detection based on immunomagnetic separation. *Analytical Chemistry*.

[40] Li D., Wang J., Wang R. (2011). A nanobeads amplified QCM immunosensor for the detection of avian influenza virus H5N1. *Biosensors and Bioelectronics*.

[41] Bilan R., Fleury F., Nabiev I., Sukhanova A. (2015). Quantum dot surface chemistry and functionalization for cell targeting and imaging. *Bioconjugate Chemistry*.

[42] Xing Y., Rao J. (2008). Quantum dot bioconjugates for in vitro diagnostics & in vivo imaging. *Cancer Biomarkers*.

[43] Montenegro J.-M., Grazu V., Sukhanova A. (2013). Controlled antibody/(bio-) conjugation of inorganic nanoparticles for targeted delivery. *Advanced Drug Delivery Reviews*.

[44] Pabst T. M., Wendeler M., Wang X., Bezemer S., Hermans P., Hunter A. K. (2017). Camelid VH H affinity ligands enable separation of closely related biopharmaceuticals. *Biotechnology Journal*.

