# Supplementary material for: Selection and Characterization of CSFV-Specific Single-Domain Antibodies and Their Application along with Immunomagnetic Nanobeads and Quantum Dots
Source: Biomed Res Int. 2020 Jan 30;2020:3201630. doi: 10.1155/2020/3201630 (PMC7013354; doi:10.1155/2020/3201630)
Supplement: Supplementary Materials — Supplementary Figure 1: expression of E2 protein with different tag proteins that were used for the selection and evaluation of single-domain antibody. Supplementary Figure 2: analysis of neutralizing activity of sdAbE2s. [file 3201630.f1.docx]

**Supplementary Material**

**Selection and characterization of CSFV-specific single-domain antibodies and their application along with immunomagnetic nanobeads and quantum dots**

Shunli Yang^1●^, Li Yuan^1●^, Youjun Shang^1^, Jinyan Wu^1^, Xiangtao Liu^1,2^, Jie Zhang^1^, Zygmunt Pejsak^3^, Katarzyna Podgórska^3^, Katarzyna Stepniewska^3^, Muhammad Umar Zafar Khan^1^, Jianping Cai^1,^^2^*, Shuanghui Yin^1^*

1. State Key Laboratory of Veterinary Etiological Biology, National Foot and Mouth Disease Reference Laboratory, Innovative Team for GI Infection and Mucosal Immunity of Swine and Poultry, Lanzhou Veterinary Research Institute, Chinese Academy of Agricultural Sciences, Lanzhou 730046, China

2. Jiangsu Co-Innovation Center for Prevention and Control of Important Animal Infectious Diseases and Zoonoses, Yanzhou 225009, Jiangsu, China

3. Department of Swine Diseases, National Veterinary Research Institute, 57 Partyzantow, 24-100 Puławy, Poland

● These authors contributed equally to this work.

* Corresponding author

Institute: Lanzhou Veterinary Research Institute, CAAS

Mailing address: Lanzhou Veterinary Research Institute, CAAS,

Xujiaping 1, Yanchangbu, Lanzhou 730046, Gansu, China.

Phone: 86-931-8342580. Fax: 86-931-8340977

Jianping Cai: E-mail: caijianping@caas.cn

Shuanghui Yin: E-mail: yinshuanghui@caas.cn

**Analysis of neutralizing activity**

The purified sdAbE2s were heat-inactivated (30 min, 56 °C) and used in a microtiter neutralization assay on Swine Testicular cells (ST). sdAbE2s, at final concentrations of 10, 20, and 100 μg/mL, were incubated with 10×tissue culture infectious dose 50 (TCID_50_) of CSFV C-strain at 37 °C for 1 h, followed by 10-times serial dilution and infection of monolayers of ST cells in 96-well plates. After another 96 h culture, the cells were examined by indirect immunofluorescence and the neutralization activity of sdAbE2s was calculated by comparing the changes of TCID_50_ value. Dilution buffer (PBS) of sdAbE2s was used as the control.

The C-strain incubated with 10, 20, 100 μg/mL of sdAbE2s showed TCID_50_/0.1 mL range from 10^3.4^ to 10^3.75^. This value was not significantly different that resulting from control incubated with PBS alone showed a TCID_50_/0.1 mL value range from 10^3.5^ to 10^3.75^ (**Fig. S2**).

**Supplementary figure caption**

**Fig. S1. Expression of E2 protein of CSFV**

The Truncated E2 protein of CSFV C-strain was sub-cloned into the expression vector *PGEX-6p-1* and *PET-32a+*, and expressed in *E.coli* (DE3). Lane 1, expression of hrE2. Lane 2, the *PET-32a+* vector control. Lane 3, purification of hrE2. Lane 4, expression of grE2. Lane 5, the *PGEX-6p-1* vector control. Lane 6, purification of grE2. Lane M, protein MW marker.

**Fig. S2. Neutralizing activity assay**

The neutralizing activity of sdAbE2s to C-strain was analyzed in ST cells. The different concentration of sdAbE2s was incubated with C-strain and the TCID_50_ value was calculated by Reed-muench methods. PBS buffer was incubated with C-strain as control. Results are expressed as the means of triplicate expriments and error bars represent ± SD.


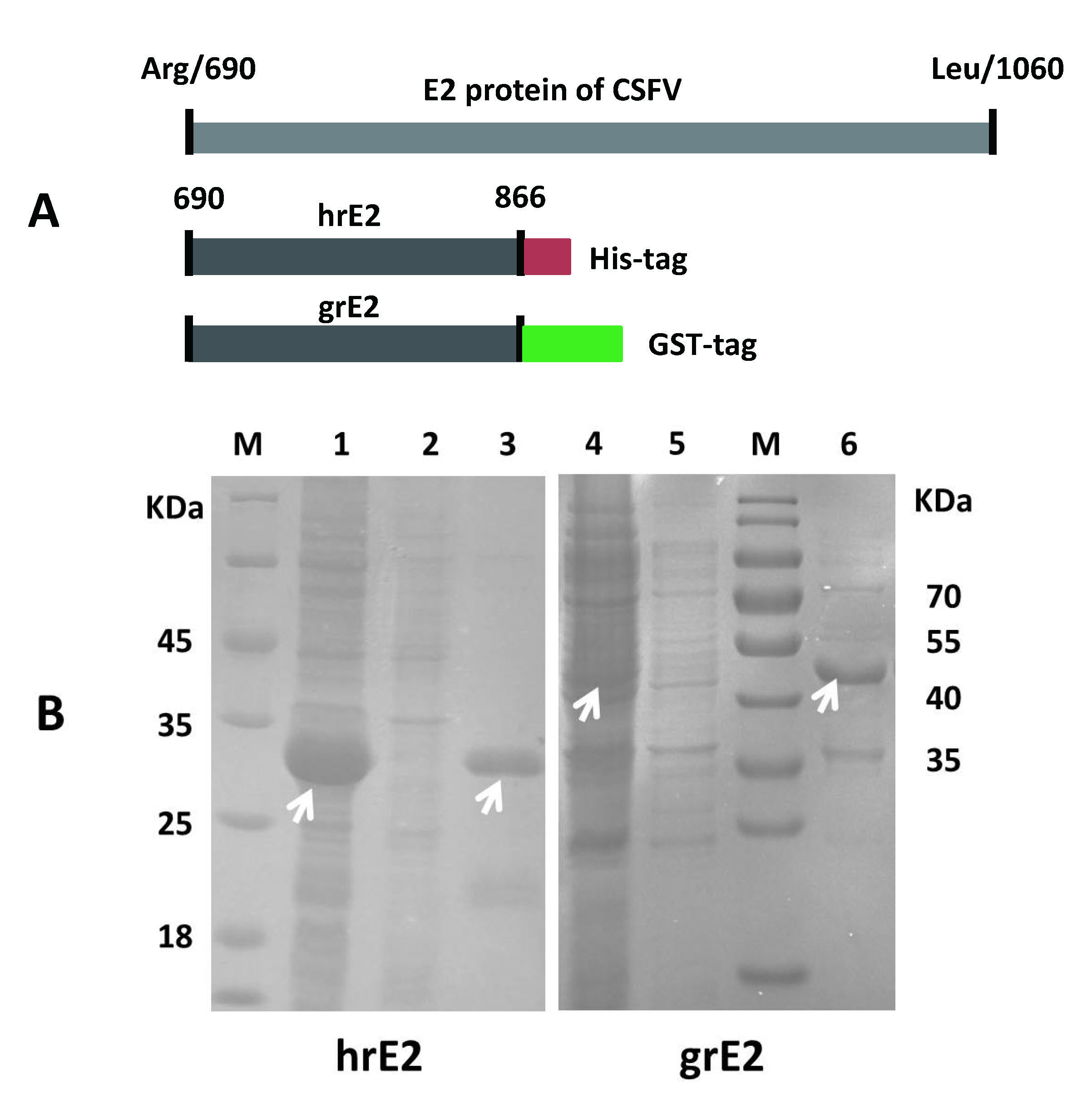


**Fig. S1**


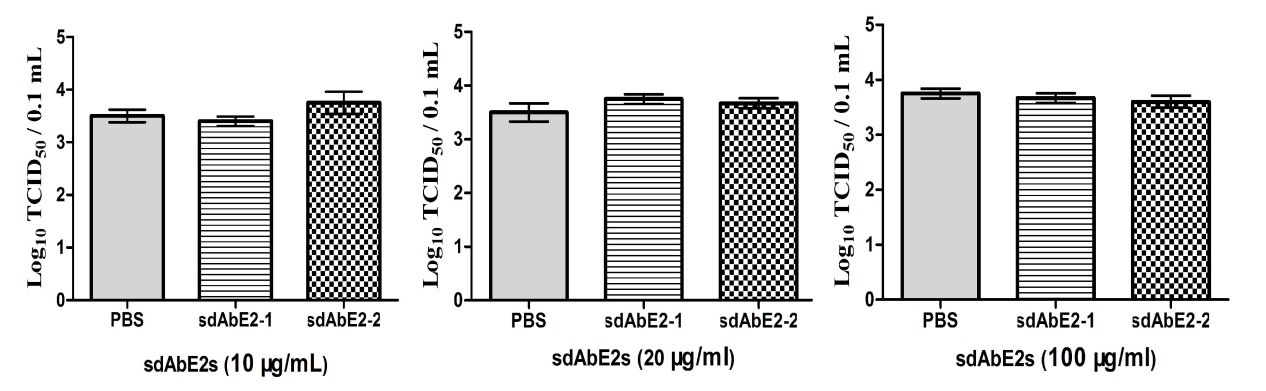


**Fig. S2**
